# Mussel *Unio douglasiae* MG from the Chihe River: mitogenome description and phylogenetic analysis

**DOI:** 10.1080/23802359.2021.1875899

**Published:** 2022-02-24

**Authors:** Danni Li, Pengjie Yang, Yunjie Zhang, Yueer Shen, Yalin Zhang, Qianxue Shao, Pengyu Chen, Shoubao Yang

**Affiliations:** a College of Life Sciences, Shaoxing University, Shaoxing, P.R. China; b Sanmen Experimental Junior High School, Taizhou, P.R. China

**Keywords:** *Unio douglasiae* MG, mitochondrial genome, phylogenetic analysis

## Abstract

The genus *Unio* is one of the widespread freshwater bivalves. To date, its intra-generic phylogeny remains controversial and therefore the mitochondrial genome data is needed. Here, we report the complete mitogenome of *Unio douglasiae* MG that is distributed in the Chihe River, a branch of Huaihe River, East China. This mitochondrial genome is 15,764 base pair in total length. It consists of 37 genes: 13 protein-coding genes, 22 tRNA genes, and 2 rRNA genes (12S and 16S). The base composition was 38.38% for A, 26.48% for T, 23.17% for C, and 11.98% for G, showing an obvious bias of higher A + T content (64.86%) than the G + C content (35.14%). Phylogenetic analysis showed that *U. douglasiae* MG is clustered with other *Unio* and *Nodularia* mollusks in the family Unionidae. These results showed that combine with morphological techniques, the mitogenome can provide useful information to further understanding of the genetics, systematics, and conservation of this endangered species.

*Unio douglasiae* (Gray) belongs to the Eulamellibranchia, Unionodae, Unio. It distributed widely in China, Korean, Eastern Russia, and Japan (Cho et al. 1983; Takaki 1992; Graf and Cummings 2007; Li et al. 2013; Nam et al. 2015; Nishio et al. 2016). In China, *U. douglasiae* is a widespread freshwater mussel distributed from the Zhujiang River, Dongting Lake, Yangtze River to Songhua River (Liu et al. 2009; Xu et al. 2009; Chen et al. 2010; Xiong et al. 2010; Xue et al. 2019). In addition, as a benthic bivalve and filter-feeder (Chen et al. 2011; Kim et al. 2011; Xiao et al. 2012; Ouyang et al. 2013; Wang et al. 2013), it plays an important role in maintaining water ecosystem balance, and serving as a biological indicator for water quality (Yokoyama and Park 2003; Watanabe et al. 2006; Maoka et al. 2012; Kim et al. 2017; Wang 2017; Jiang et al. 2020). However, its wild population declines rapidly due to overfishing, water pollution, and destruction of habitats.

In this study, the *U. douglasiae* MG specimen was collected from the Chihe River, a branch of Huaihe River in Mingguang city, Anhui province of China (Latitude 32.789726 and longitude 117.969780), and has been deposited at the Aquatic Service Platform of Shaoxing city (Voucher no. SXAF20200710).

The complete mitogenome sequence of *U. douglasiae* MG is obtained by PCR amplification and Sanger sequencing using an ABI3730 sequencer (Applied Biosystems, USA). It’s 15,764 bp in total length (GenBank accession no. MT764726), including 37 genes: 13 protein-coding genes, 22 tRNA genes, and 2 rRNA genes (12S and 16S). The base composition was 38.38% for A, 26.48% for T, 23.17% for C, and 11.98% for G, showing an obvious bias of higher A + T content (64.86%) than the G + C content (35.14%).

Similar to other mollusks, two tRNA genes (*tRNA*^Asp^, and *tRNA*^His^) and nine protein-coding genes (*COXI*, *COXII*, *COXIII*, *ND3*, *ND4*, *ND4L*, *ND5*, *ATP6*, and *ATP8*) are encoded on the light strand (L-strand). Typical ‘ATG’ is used as the initiator codon in eight protein-coding genes (*COXII*, *COXIII*, *ND2*, *ND3*, *ND4*, *ND4L*, *ND6*, and *ATP6*). ‘TAA’ is used as the terminator codon in eight protein-coding genes (*COXII*, *COXIII*, *ND2*, *ND3*, *ND5*, *ND6*, *ATP6*, and *Cytb*), and ‘TAG’ is used in five protein-coding genes (*COXI*, *ND1*, *ND4*, *ND4L*, and *ATP8*).

The total length of two rRNA genes (12S and 16S) is 2152 bp, and they are separated by three tRNA genes including *tRNA*^Lys^, *tRNA*^Thr^, and *tRNA*^Tyr^, which is identical to the rRNA gene arrangement of other mussels (Breton et al. 2011; Wang et al. 2016a, 2016b).

Twenty-six complete mitogenome sequences were used for phylogenetic tree construction by the neighbor-joining method (Figure 1). The results showed that *U. douglasiae* MG is clustered with other *Unio* and *Nodularia* mollusks in the family Unionidae. The present study shows that, combine with morphological techniques (Wei et al. 1994; Wu et al. 1999), the mitogenome can provide useful information to further understanding of the genetics, systematics, and conservation of this endangered species.

**Figure 1.**
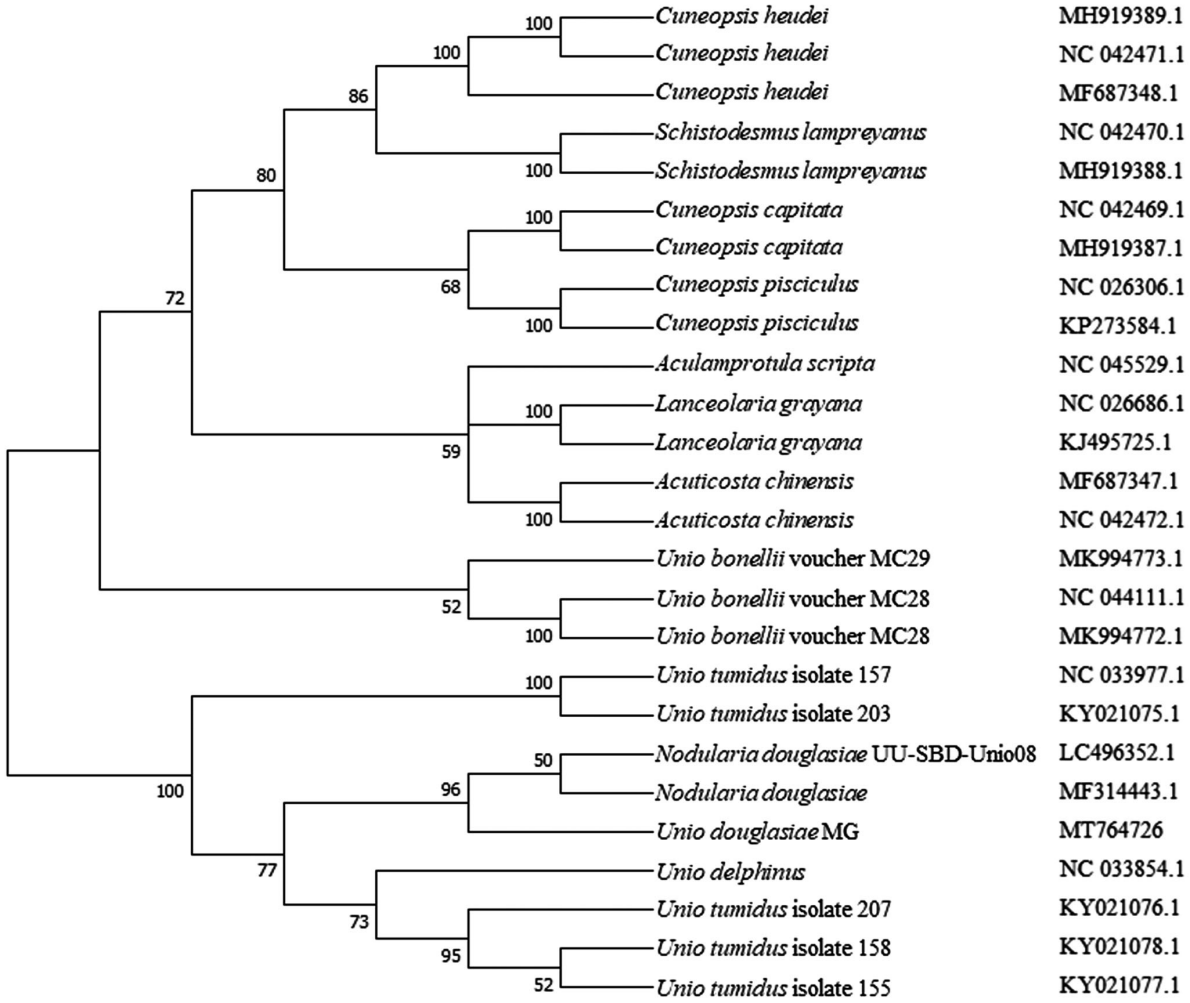
The phylogenetic analysis of *Unio douglasiae* MG and other mollusks based on the mitogenome sequences.

## Data Availability

The data that support the findings of this study are freely available at NCBI GenBank database (https://www.ncbi.nlm.nih.gov) with a accession no. MT764726. And the data that support the findings of this study are also available from the corresponding author, Dr. Yang, upon reasonable request.
